# Strategic Medical Student Engagement Enhances Knowledge, Interest, and Recruitment in Radiation Oncology

**DOI:** 10.7759/cureus.104928

**Published:** 2026-03-09

**Authors:** Lisa A McGee, Jonathon Ashman, Terence Sio, Randa Tao, Joshua Niska, Richard Kunath, Brady S Laughlin

**Affiliations:** 1 Radiation Oncology, Mayo Clinic Arizona, Phoenix, USA

**Keywords:** medical student education, medical student engagement, radiation oncology, radiation oncology awareness, residency recruitment

## Abstract

Background

Residency match data suggest there may be a suboptimal number of radiation oncology (RO) residency applicants. This report describes one RO residency program’s approach to medical student engagement (MSE) to improve interest in RO and enhance recruitment.

Methods

One RO residency program implemented a strategic approach to MSE. Key components of MSE were identified and included social media, didactic lectures, open house events, and research mentorship. Events that led students to conduct clinical rotations, preclinical observerships, research rotations, or residency applications were considered measures of success.

Results

Seven Instagram posts and 14 X posts were created to promote MSE events and announce departmental achievements. Didactic lectures were offered to all five local medical schools, three of which participated. One medical school included an RO didactic lecture during the MS2 oncology block, and two medical schools incorporated student interest group lectures. Virtual and in-person open house events were conducted to educate students on RO. Notably, 4 out of 10 attendees expressed interest in RO after the open house by completing observerships and initiating research projects (N = 2), requesting a future student interest group RO lecture (N = 1), and completing a rotation and applying to residency the following academic year (AY) (N = 1). Four students established a formal research mentorship. One student completed a poster presentation at a national meeting, wrote four manuscripts, and was accepted to medical school. Another student completed a poster presentation at a national conference. The remaining two students have projects in process. All four students completing research projects maintain an interest in RO.

Conclusions

Implementation of a comprehensive MSE initiative by an individual residency program is feasible. Early results suggest improved interest in the specialty, which can lead to enhanced residency recruitment.

## Introduction

Over the last five years, resident physician trainees have entered the field of radiation oncology (RO) through an increasingly broad range of educational and training pathways. Despite this, National Resident Matching Program (NRMP) data trends over the last five years suggest a suboptimal number of RO residency applicants compared to open residency positions. Applicant numbers for open advanced residency positions at the PGY2 level, which represents the majority of RO programs, show a relatively stable number of applicants per open position, with a mean of 1.13 (range: 1.01-1.34) [1-5]. While the applicant-per-open-residency-position ratio has hovered around 1.13 in recent years, many residency program positions have not been filled through the initial NRMP match process. During the years 2021-2023, there were 28, 26, and 24 positions available, respectively, in the Supplemental Offer and Acceptance Program (SOAP), which represented 13%-16% of the open advanced PGY2 residency positions [1-3]. The number of positions offered through the SOAP improved in 2024 to nine positions (5%) and in 2025 to three positions (2%) [4,5]. The decrease in the number of open positions offered in the SOAP is promising over the last two years. However, the data overall suggest that there is still a need for our specialty to improve and stabilize the overall number of qualified residency applicants.

Published data demonstrate that medical student engagement (MSE) activities can improve student knowledge of RO and recruitment outcomes [6]. However, there is scarce data documenting a comprehensive MSE program and its success in establishing a pipeline of qualified medical student applicants to the specialty or to specific RO residency programs. This report describes one residency program’s approach to implementing a formal MSE initiative, with the goal of increasing medical students’ interest in the specialty and their application to the RO residency.

## Materials and methods

A comprehensive MSE program was developed and implemented by our RO residency program for the 2024-2025 academic year (AY), with the goal of educating medical students on basic radiation medicine, garnering interest in the field of RO, and enhancing residency program recruitment. In July 2024, a new MSE Committee was created and composed of six faculty members from the Education Committee, including the Residency Program Director, the Medical Student Clerkship Co-directors, two faculty at large, and one resident physician. The committee held monthly meetings to identify opportunities for MSE, conduct outreach, and plan events.

Several areas of MSE opportunities were identified, which included social media outreach, didactic lectures, virtual and in-person open house events, and research mentorship. One faculty member was appointed to social media efforts and was tasked with creating social media posts to promote the residency program on both Instagram and X platforms. The remainder of the team combined efforts to contact all five local medical schools to schedule RO lectures during the standard oncology curriculum or with student interest groups. Additionally, the committee planned both a virtual open house conducted via Zoom as well as an in-person open house hosted within our department. Four faculty members mentored four students on research projects. Other MSE events included an American Society of Therapeutic Radiation Oncology (ASTRO) dinner, one panel discussion at a local medical school, a poster walk with medical students, and participation via a Radiation Oncology Virtual Education Rotation (ROVER) event. All events focused on basic RO education and highlighted the benefits of working in the specialty, including longitudinal patient relationships, innovation of cancer treatment and evolution of technology, a collaborative work environment with a multidisciplinary approach to patient care, favorable work-life balance, and a stable job market.

The goal of this initiative was to extend MSE efforts beyond what has been traditionally performed in our department. We, therefore, did not include clinical rotations or preclinical observerships, since all rotators for the 2024-2025 AY sought out rotations in our department independently and did not result from our MSE efforts. Events that led students to conduct clinical rotations, preclinical observerships, research rotations, or residency applications were counted as a measure of success for the MSE initiative.

## Results

Social media

In total, seven Instagram posts were created to promote the RO residency program. The posts promoted topics such as advertising MSE events, including the department-hosted ASTRO dinner for medical students, the virtual open house, and the in-person open house to showcase our department. The announcement for the in-person open house was the most popular and resulted in 2,325 views and 53 likes. Additionally, group photos from national conferences and a visiting professor were shared. Lastly, we shared Match results announcing our newly matched resident in March 2025, which resulted in the largest number of views (4,577) and likes (93). Over the course of the AY, we saw a trend of an increased number of views and likes over time (Table 1).

**Table 1:** Social Media Posts on Instagram ASTRO, American Society for Radiation Oncology; PTCOG NA, Particle Therapy Cooperative Group North America

Date of Post	Post Content	Likes	Views	Interactions	Profile Activity
September 11, 2024	ASTRO Dinner Advertisement	11	673	11	2
September 12, 2024	Virtual Open House Advertisement	23	796	24	6
September 30, 2024	ASTRO Group Photo	64	1,099	64	6
November 21, 2024	PTCOG NA Group Photo	62	1,366	63	2
November 25, 2024	Visiting Professor	44	1,956	44	21
December 3, 2024	In-Person Open House	53	2,325	56	16
March 21, 2025	Residency Match 2025	93	4,577	112	99
Not Applicable	Median	55	1,366	56	6

There were 14 social media posts created for the X (formerly Twitter) platform. These posts also promoted comparable topics, including advertising student outreach events (N = 4), faculty achievements, such as speaking and research initiatives, board certification (N = 9), as well as announcing our residency program’s match results (N = 1). Popular posts included the Match announcement, garnering 2,118 impressions and 30 likes, as well as a board certification announcement, resulting in 1,238 engagements and 36 likes. Again, over the course of the AY, we saw an overall trend of an increased number of likes, impressions, and engagements over time (Table 2).

**Table 2:** Social Media Posts on X ASTRO, American Society for Radiation Oncology; ABR, American Board of Radiology

Date of Post	Post Content	Likes	Retweets	Impressions	Engagements
September 11, 2024	ASTRO Dinner Advertisement	4	1	447	17
September 12, 2024	Virtual Open House Advertisement	5	2	456	33
September 26, 2024	ASTRO Dinner Advertisement (repeat)	5	1	839	33
November 16, 2024	Faculty Speaker at National Conference	14	1	500	Not Available
November 22, 2024	Faculty Research Project	26	8	1,373	82
November 22, 2024	Faculty Speaker at National Conference	8	1	394	31
December 3, 2024	Open House Group Photo	22	0	944	60
December 13, 2024	Faculty News Feature	5	1	327	11
December 13, 2024	Faculty Speaker at International Conference	6	1	294	18
February 7, 2025	Faculty Research Project	17	9	1,662	68
March 11, 2025	Faculty Manuscript Publication Announcement	16	3	1,509	112
March 11, 2025	Faculty Manuscript Publication Announcement	3	2	439	Not Available
March 25, 2025	Residency Match 2025	30	4	2,118	75
April 25, 2025	Faculty ABR Board Certification Announcement	36	0	1,238	169
Not Applicable	Median	11	1	670	47

Educational lecture events

The initial goal was to lecture at each of the five local medical schools in some capacity. Committee members reached out to contacts at all five of the medical schools and requested lecture time with medical students. One medical school included an RO faculty lecturer in the MS2 curriculum, teaching an overview of RO and the management of patients requiring emergent radiation. We also contacted three student interest groups at this same medical school, none of which were interested in scheduling a lecture. At the second medical school, three student interest groups were contacted, and only one of these scheduled a lecture. At a third medical school, we contacted one student interest group that scheduled a lecture, and invited members from two additional student interest groups. A fourth medical school did not respond to our offer to schedule lectures. Despite numerous contacts within our affiliated medical school, we were unable to secure lecture time due to a lack of student interest (Table 3).

**Table 3:** Medical Student Engagement Event and Lecture Schedule Description: IGNITEMED is the name of a women's medical student association. ASTRO, American Society for Radiation Oncology; ACR, American College of Radiology; AZ, Arizona; MS, Medical Student; ACRO, American College of Radiation Oncology; ROVER, Radiation Oncology Virtual Education Rotation

Date	Presenter	School/Event	Student Participants (If Known)
September 19, 2024	4 faculty, 3 residents	Virtual Open House	15
September 28, 2024	5 faculty, 2 residents	ASTRO Dinner	9
October 12, 2024	1 faculty	ACR Interest Group, All AZ Medical Schools	50
November 1, 2024	1 faculty	MS 2 Lecture at Local AZ Medical School	8 in-person attendees, recorded for the whole MS 2 class
November 14, 2024	1 faculty	Local AZ Medical School student interest clubs: Oncology, Radiology, and Interventional Radiology	Not Available
December 2, 2024	5 faculty, 3 residents	In-Person Open House	10
January 24, 2025	1 faculty	IGNITEMED Panel Discussion	Not Available
March 2025	1 faculty	ACRO Poster Walk for Medical Students	Not Available
April 4, 2025	1 faculty, 3 residents	ROVER	Interested medical students throughout the country

We hosted a virtual open house via Zoom. Virtual open house invitations were sent to all in-state medical schools, as well as advertised on Instagram and X, targeting medical students from all years of training. The virtual open house included a general RO residency overview presentation delivered by one of our resident physicians, followed by a question-and-answer session (Table 3). In total, 16 students attended this event; 15 of these students applied for residency during the 2024-2025 AY. Three students completed a clinical rotation during the same AY, and two students completed an observership or on-site meet-and-greet.

The in-person open house was conducted on-site in our department (Table 3). All in-state medical students from any year of training were welcome, and email invitations were sent via contacts at each of the six in-state medical schools. This event started with a lecture giving an overview of the RO residency, an introduction of faculty and residents - each providing an individual testimonial about why they chose the field of RO - general concepts of RO, a departmental tour in which students were allowed to see the treatment machines, and, finally, a question-and-answer session over dinner. Of the 10 students who attended the in-person open house, one student completed an MS4 audition rotation and applied to our residency program during the following 2025-2026 AY. Two students pursued both preclinical observerships and research rotations, one of whom received an ASTRO Medical Student Fellowship Award. Another student contacted our team during the 2025-2026 AY to schedule a lecture for a student interest group at his medical school.

Our department hosted a pizza dinner for medical students attending the national ASTRO meeting, who were interested in meeting representatives from our residency program (Table 3). In total, nine students, five faculty, and two residents attended this event. Of these, eight students were eligible to apply to our residency program; all eight applied for our residency program during the 2024-2025 AY.

Student research mentorship

During the 2024-2025 AY, four students received formal oversight and mentorship from different faculty while working on research projects. One of the premedical students produced one poster presentation at a national meeting and four manuscripts during this period, was accepted to medical school, and maintains an interest in potentially pursuing RO as a specialty upon graduation. Another premedical student completed a poster presentation at a national meeting and maintains an interest in RO if accepted to medical school. Two medical students established a formal research mentor following the in-person open house and are currently still working on the completion of their projects.

## Discussion

Our approach is unique because it comprehensively includes multiple avenues of MSE, with the goal of encouraging medical students to learn more about RO and potentially pursue RO residency. Currently, we employ several educational approaches, striving to increase exposure to the field, including social media, virtual and in-person programs, structured lectures, and research rotations. To our knowledge, this report of our experience is the first to highlight a proactive approach for a residency program to increase medical student exposure and enhance recruitment. This comprehensive approach to MSE is scalable and can be easily reproduced by other programs, either in part or as a whole program.

Current published data suggest that outreach early in medical school, via live events and specialty-specific mentorship, is successful in improving residency recruitment. A Canadian summership program, consisting of clinical exposure, research, and mentorship over a six-week period, presents compelling career conversion data, with 30.5% of participants entering RO compared to 0.7% national match rates [6]. Additionally, it has been reported that there was maintained or increased interest in radiation oncology among those awarded the Canadian Association of Radiation Oncology Annual Scientific Meeting Medical Student Research and Mentorship Award [7]. It has also been reported that mentorship of medical students in the field of RO led to match rates of 29.3% of mentees, which is higher compared to national match rates [8]. 

RO educational lectures, particularly within the first two years of medical school, have demonstrated promise in motivating medical students to pursue additional information about the RO specialty and to pursue RO as a career. A radiation oncologist-led oncology block at a single institution demonstrated favorable student perceptions since its inception [9,10]. Additionally, first- and second-year students exposed to multidisciplinary tumor board discussions reported a better educational experience compared with traditional shadowing [11]. A third-year clinical oncology elective led by RO over 10 years found that 15% of participants decided on oncology, with 74% choosing RO [12]. Implementing a formal medical student curriculum into RO rotations has also been demonstrated to improve the RO knowledge base. The Radiation Oncology Education Collaborative Study Group has developed three core lectures (Overview of Radiation Oncology, Radiation Biology/Physics, and Practical Aspects of Simulation/Radiation Emergencies) and a treatment planning workshop [13,14]. Knowledge improved significantly in students completing the course (63.9%-80.2%, p < 0.01). At six months post-rotation, there was a trend toward increased retention of knowledge in those completing the course (70.5% vs 65.6%, p = 0.11) [14]. National trends demonstrated higher adoption of structured didactics within medical student clerkships, up to 43.2% in 2018, from 28.3% in 2013 [15].

Virtual education programs have also demonstrated that they are another effective way to engage medical students interested in the field of RO. ROVER provides case-based lectures and discussions across multiple disease sites with representatives, including faculty and residents from multiple institutions. The program demonstrated improved knowledge of the role of RO in over 200 students across multiple cancer types (p < 0.001) [16]. Furthermore, a two-week in-person and virtual curriculum, with contouring modules, has also shown increased comfort with the field [17].

Targeted outreach, specifically for students underrepresented in medicine (URiM), has demonstrated positive outcomes. One pilot study found that, following the incorporation of RO into one of these early pathway programs, nearly 70% of participants reported increased interest in the field [18]. A program that offered virtual lectures to medical schools without RO departments, and had high enrollment of URiM students, found that interest in the field increased regardless of gender, race, or ethnicity [19].

Social media use by residency programs has increased in recent years and is commonly used by both residency applicants and residency programs [20-29]. Medical student survey data demonstrate that social media influences both perceptions and the ranking of residency programs [29]. Social media can be used to share information regarding a residency program, such as resident experience, program and departmental accomplishments, and program highlights [26,27]. Social media can also be used to advertise MSE events [26]. Candidates can use social media to directly connect with a program representative to obtain additional program information and seek mentorship or advice [26]. Some programs even offer virtual electives [26]. While social media can be effective at generating RO and residency program interest and is a convenient forum for information sharing, it is uncertain what impact our program's social media presence has on motivating students to complete clinical rotations, pursue research electives, or apply to an RO residency in our department. Additionally, development of social media content can be time-consuming and is unlikely to completely replicate an in-person experience [26,28].

Cumulatively, the current evidence therefore supports the notion of having a multifaceted approach to MSE, as utilized by our residency program. We have incorporated in-person and virtual exposure options, specialty-specific research mentorship, and RO educational opportunities for medical students, in addition to our pre-existing immersive experiences of clinical rotations and preclinical observerships. We have primarily focused our efforts on local medical students and recognize the importance of having an inclusive outreach effort to all medical students nationally. We have focused on MSE activities geared toward students across all levels of medical school training and have included a couple of premedical students in our MSE efforts. All MSE efforts focused on basic RO education and highlighted the many benefits of a career in RO, including longitudinal patient relationships, innovation and technology, a multidisciplinary work environment, a favorable work-life balance, a competitive salary, and a stable job market.

Our limited data show that the in-person open house and RO research mentorship were the two most productive avenues of MSE, resulting in students pursuing additional educational opportunities (40% of in-person open house attendees) and maintaining an interest in pursuing RO as a career (100% of students having research mentorship). In our experience, the ASTRO dinner and virtual open house did not generate new interest in RO among attendees. The attendees at both events were already applying to RO residency, and these efforts were therefore more useful for current recruitment efforts rather than developing a pipeline of future applicants.

While the early results of our MSE program are promising, several limitations are noted. First, a longer follow-up is needed to identify the impact longitudinally on RO knowledge and residency recruitment in the cohort of medical students exposed to these efforts. Participants in MSE events need to be continuously tracked and compared to recruitment outcomes annually. Second, the true impact of our educational lecture efforts remains unknown since it was not possible to track the specific attendees from other institutions. To understand the true impact of our lecture efforts, we would need to obtain attendance records for these lectures and could then track the impact over time.

Third, we recognize the importance of broader MSE and need to incorporate more efforts to include medical students nationally. Collaboration of efforts with other residency programs or nationally, with the assistance of ASTRO, could help build more extensive MSE initiatives. Fourth, we did not specifically focus our efforts on any particular demographic of medical students, although we recognize that this could potentially be accomplished by intentionally targeting more diverse medical student populations.

Lastly, we do not currently have a useful way to correlate the impact of our program's social media posts with the success of MSE efforts within our program. Social media templates and specific targeting criteria could be used, and a routine posting schedule could be implemented. Future efforts could include additional tracking methods for social media, such as links for attendance confirmation, mentorship request forms, and medical student surveys to quantify specialty interest. Additionally, time and resource investment could be tracked, and automated tools could be used to improve efficiency and impact.

## Conclusions

These descriptive data establish a feasible, comprehensive approach for a residency program to initiate medical student outreach at all levels of medical student training, including structured lectures, open house events, targeted social media, and longitudinal research mentorship. Because RO is not a standard component of the medical student curriculum, outreach programs are essential to generate medical student interest and engagement in the specialty. Over time and with additional follow-up, these efforts may strengthen the residency applicant pipeline and produce better alignment between future resident and program training goals, as well as specialty demands. Future controlled studies are needed to verify these outcomes on a larger scale.
